# What Do Adolescents Know About One-Health and Zoonotic Risks? A School-Based Survey in Italy, Austria, Germany, Slovenia, Mauritius, and Japan

**DOI:** 10.3389/fpubh.2021.658876

**Published:** 2021-03-30

**Authors:** Paolo Zucca, Marie-Christin Rossmann, Mitja Dodic, Yashwantrao Ramma, Toshiya Matsushima, Steven Seet, Susanne Holtze, Alessandro Bremini, Ingrid Fischinger, Giulia Morosetti, Marcello Sitzia, Roberto Furlani, Oronzo Greco, Giulio Meddi, Paolo Zambotto, Fabiola Meo, Serena Pulcini, Manlio Palei, Gianna Zamaro

**Affiliations:** ^1^Central Directorate for Health, Social Policies and Disabilities, Friuli Venezia Giulia Region, Trieste, Italy; ^2^Biocrime Veterinary Medical Intelligence Centre, c/o International Police and Custom Cooperation Centre, Thörl-Maglern, Austria; ^3^Agriculture, Forestry, Rural areas Veterinary Department, Land Carinthia, Klagenfurt, Austria; ^4^Private Practitioner, Kozina, Slovenia; ^5^Mauritius Institute of Education, Réduit, Mauritius; ^6^Hokkaido University, Sapporo, Japan; ^7^Leibniz Institute for Zoo and Wildlife Research, Berlin, Germany; ^8^South Tyrol Health Department, Veterinary Services Bolzano, Bolzano, Italy; ^9^Italian Financial Police, Regional Command Friuli Venezia Giulia Region, Trieste, Italy; ^10^SCIP International Service of Police Cooperation, International Police and Custom Cooperation Centre, Thörl-Maglern, Austria; ^11^Veterinary Services, Bolzano, Italy; ^12^Area Science Park, Padriciano, Italy

**Keywords:** one health, zoonoses, rabies, risk perception, adolescents, education, biocrime project, medical intelligence

## Abstract

More than 60% of the 1,700 infectious diseases that affect human come from animals and zoonotic pandemics, after starting from sporadic phenomena limited to rural areas, have become a global emergency. The repeated and frequent zoonotic outbreaks such as the most recent COVID-19 pandemic can be attributed also to human activities. In particular, the creation of enormous intensive domestic animal farms, the indiscriminate use of antibiotics, the destruction of forests, the consumption of the meat of wild animals and the illegal animal trade are all factors causing the insurgence and the transmission of zoonotic diseases from animals to humans. The purpose of this study was to explore the knowledge of the One Health concept including the zoonotic risk potentially derived from illegally traded pet animals and wildlife among adolescents in 6 different countries (Italy, Austria, Slovenia, Germany, Mauritius, and Japan). A representative sample of 656 students was recruited and all participants took an anonymous questionnaire. Data were analyzed by ANOVAs to estimate the prevalence of correct health prevention behaviors and to identify the influential factors for these behaviors. After two theoretical-practical lectures, the same anonymous questionnaire was administered for the second time in order to assess the efficacy of the program. The proportion of students who did not know that many diseases affecting humans come from animals is 28.96% while 32.16% of them did not know what a zoonosis is. The circularity of the One Health concept related to the transmission of diseases from animals to humans and vice-versa is not understood from a large prevalence of the adolescents with 31.40 and 59.91% of wrong responses, respectively. Furthermore, rabies is not considered as a dangerous disease by 23.02% of the adolescents. After two theoretical-practical classroom sessions, the correct answers improved to 21.92% according to the different question. More than a third of the student cohort investigated showed a profound ignorance of the zoonotic risks and a poor understanding of the One Health concept. The authors believe that the teaching of health prevention with a One Health approach and a practical training should be included in every school curriculum.

## Visual Version

Cultured people read all day, mostly unconsciously observing street signs, glancing at product labels in shops, menus etc. These forms of reading tend to be superficial and short-lived but there are times when we also read complex texts with greater intensity and duration (1, 2). However, the world of the screen and the internet are very different places from the page of a book. How do Internet users read? Well, in summary they don't read [Nielsen (3) cited by Carr (2)]. Probably, no reader will print this article on paper, no daredevil reader who will reach the end of reading this article will do so without being interrupted countless times and in most cases those who will view the article in its open-source form, reading it from their desktop monitor or more likely from their mobile phones, they will spend an average of 19–27 s looking at the page and then moving on to the next one. For these reasons, we have decided to create also a visual version in infographic format of the article to be associated with it, to prevent our intelligence from flattening out on artificial intelligence, as in Hal 2001 by Kubrick [cited by Carr (2)] within a process of emotional desertification. The paper version is dedicated to your cerebral cortex while the infographic ones will please your amygdala. The first requires time and concentration; the second requires an emotional involvement and a willingness to accept new challenges. The choice is yours but, in any case, thank you for your attention.

## Introduction

A zoonosis (from the Greek word: ζῷoν zoon “animal” and νóσoς nosos “disease”) is any disease or infection that can be transmitted from vertebrate animals to humans. More than 60% of the 1,700 infectious diseases that affect humans come from animals like the Sars-Cov-2, Ebola, Hiv, SARS, MERS, Swine, and Avian flu, Zika, etc. pandemics, after starting from sporadic phenomena limited to rural areas, have become a global emergency. Emerging zoonoses are a growing threat to global health and have caused huge economic damage in the past 20 years because they have important impacts on public health, livestock economies, and wildlife conservation (4–8). In addition to being a public health problem, many of the zoonoses are not just problems confined to remote areas but are serious threats to global public health (9–12).

**Figure 1:**
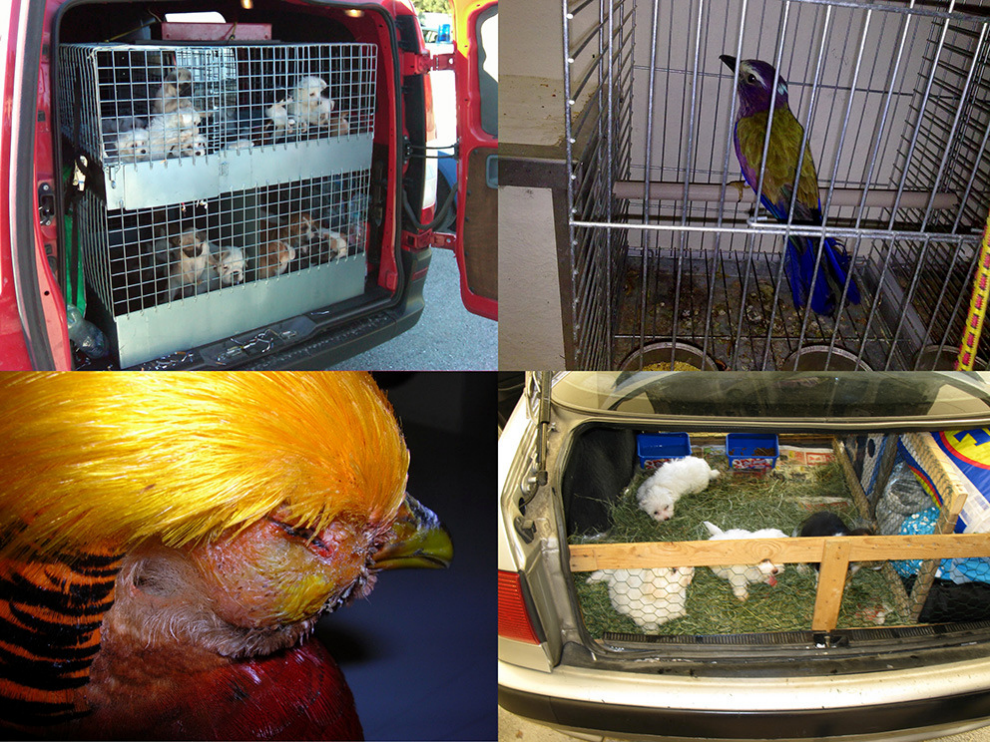
Although major hotspots of mammal hosts occur in the New and Old World tropics (South America and Eastern Africa, particularly) more zoonoses are concentrated in northern latitudes, Eastern Africa, and Southeast Asia (13). According to these findings, it is very important to emphasize that zoonoses are not “exotic diseases” for North American and European citizens (16). In fact, zoonotic agents such as Toxocara spp., Chlamydia psittaci, Salmonella spp., Giardia spp., and other viral and bacterial zoonotic diseases are routinely diagnosed among pets from illegal animal trade (16) sold to unwitting parents of children and adolescents (Images credits Paolo Zucca).

The repeated and frequent outbreak of pandemics can also be attributed to human activities. In particular, the creation of enormous intensive domestic animal farms, the indiscriminate use of antibiotics on intensive breeding farms, the destruction of forests, the consumption of the meat of wild animals (bush meat) and the illegal animal trade are all factors contributing to the insurgence and the transmission of zoonotic diseases from animals to humans (7, 13–15). Many people think that zoonoses are a marginal health problem in economically advanced countries since their spread is minimal or non-existent. This is a misperception since it has been shown that the risk of contracting a zoonotic disease from terrestrial mammals, on the contrary, is higher in North America, Europe and Asia than in South America and Africa (Figure 1).

The frequency with which new infectious diseases are emerging (Emerging Infectious Diseases), especially zoonoses, underscores the necessity of shifting from a reactionary to a pre-emptive approach to mitigating infectious disease (17). Preventing the spread of a zoonosis in the human population is more efficient the faster the spread is detected, as “early detection” and rapid control measures reduce disease incidence in humans and animals (6). The early detection mechanism was historically based on the health monitoring activities of international and national public health services, with the collaboration among veterinarians, physicians, and diagnostic laboratories.

With the development of medical and open-source epidemiological intelligence, the classical mechanism of “early detection” has been implemented and enhanced with additional tools that are not limited to monitoring but seek to extend the concept of early detection to that of “*early prediction*.” Although, as the Nobel Prize winner for physics Niehls Bohr said, “it is difficult to make predictions, especially those about the future,” the early prediction systems thanks to the work of computer scientists, statisticians, epidemiologists, veterinarians, physicians, and other professionals with transversal skills provide predictions fairly accurately in probabilistic terms on the times and places of onset of possible future outbreaks and spillovers. For instance, on December 30 of 2019, an artificial intelligence platform created by BlueDot Inc., a Canadian software company, identified a cluster of “unusual pneumonia” cases happening around a market in Wuhan, China, and flagged it 9 days before the World Health Organization (WHO) released its statement alerting people to the emergence of a novel coronavirus (18, 19).

**Figure 2:**
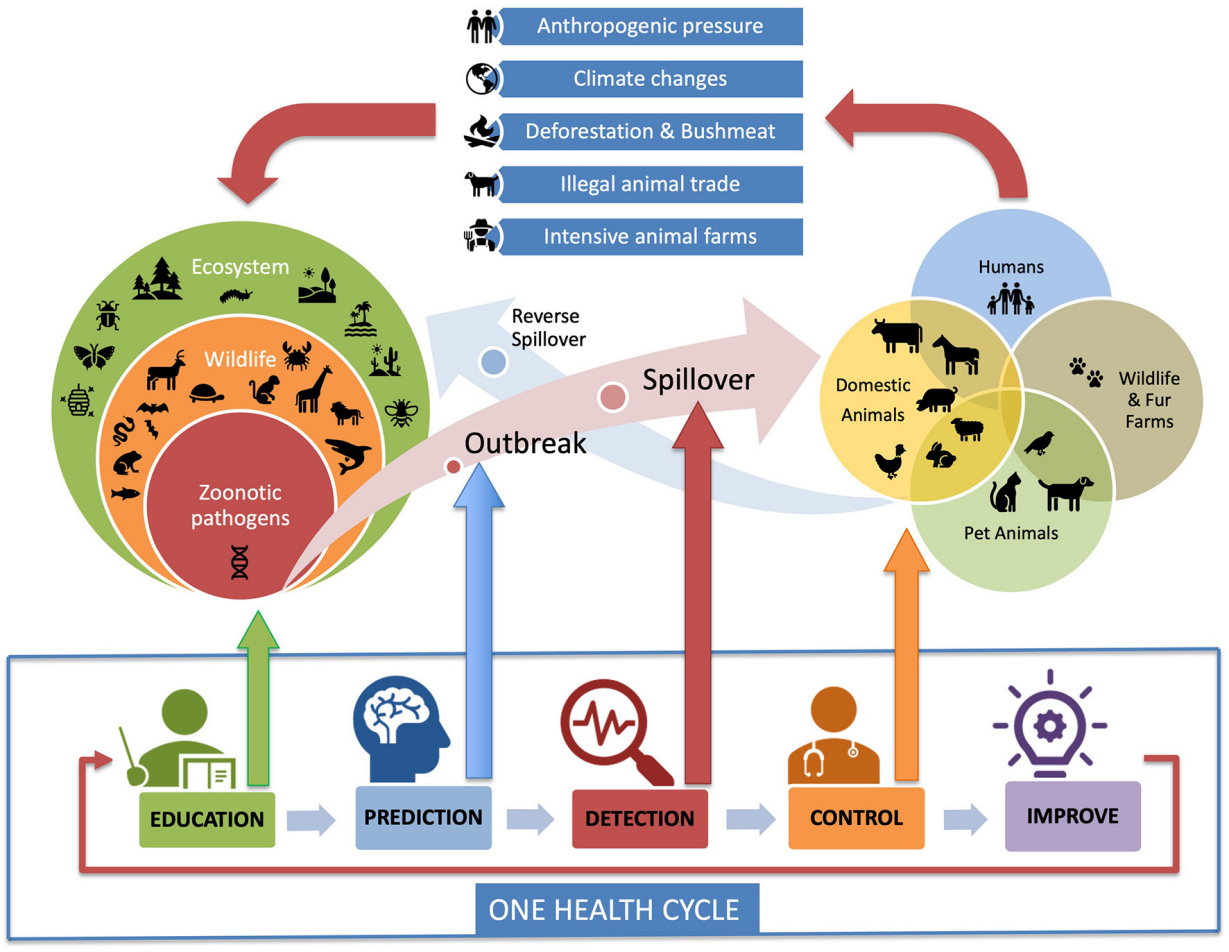
The One Health Cycle explains how the management of a zoonotic pandemic with a One Health approach works. A zoonotic pathogen from wild animals moves into livestock, pets, fur animals, or directly to humans. The One Health approach introduces the processes of “early education” (green) and “early prediction” (blue) that boost the early detection (red) and control (orange) efforts. Early education increases the number of young people able to recognize a zoonosis, while the Artificial Intelligence systems used in early prediction increase the ability to predict an outbreak in wildlife before the spillover can occur. Anthropogenic pressure, deforestation and bushmeat, climate changes, illegal animal trade, and intensive animal farm are all human-generated causal factors causing the emergence and resurgence of zoonotic diseases (Image credit Paolo Zucca).

Despite the great development of knowledge in the field of early detection and early prevision, a human limitation of this disease prevention system continues to exist, consisting of the fact that there are few people involved in early detection and forecasting compared to the population worldwide while the frequency with which new infectious diseases are emerging is increasing. The mechanisms underlying early detection and early prediction, despite the diagnostic capacity of the laboratories and the processing capacity of Artificial Intelligence (AI), need someone to feed the system by examining individual cases of disease and reporting the highest number of single outbreaks for it to work properly. In summary, we need to extend the audience of observers and this can only happen by starting with the education and involvement of young people. Therefore, to the initial concept of early detection, to which the early prediction mechanism was subsequently associated, it is necessary to insert a third mechanism that extends the audience of potential figures capable of correctly perceiving biological risk, avoiding it and therefore quickly reporting the onset of a potential zoonosis in the population to the other two systems. This mechanism that involves a much more widespread audience of people awareness to biological risk issues can be defined as “*early education*” (Figure 2).

### The One Health Cycle

The origin of the theoretical framework of the integration mechanism of education prediction, alert, control and improve to successfully prevent and control disease outbreaks belongs to the concept of One Health. One Health is defined as a cooperative, multisectoral and interdisciplinary approach that operates at a global, national, regional and local level, the aim is to improve human health by monitoring the human-animal-environment interface (20–23). This approach sees the health of humans, animals and ecosystems as an interconnected network, rather than problems to be tackled individually. Key concepts of One Health include viewing the health of all species as needing to be balanced, focusing on health assessment and disease prevention rather than exclusively on treatment and promoting a strong collaborative endeavor between human and veterinary medicine (16, 20, 23). Indeed, employing a pragmatic, preventative One Health approach to endemic zoonoses has been proposed to be both more equitable and has more effective benefits, compared to exclusively treating human cases of disease (21). It is quite challenging to describe exactly the application of the One Health approach to a zoonotic spillover control process because One Health is based on a very broad and interdisciplinary theoretical framework. A pathogen control process is a series or set of activities that interact to produce benefits to people and animals. If the spillover management activities are considered in conjunction with the One Health Cycle, it becomes easy to understand how the One Health approach integrates and can optimize the spillover management process (Figure 2).

Anyone can get sick from a zoonotic disease, including healthy people and even those with pre-existing conditions. Indeed, some people are more at risk than others and these groups of people include children, people with weakened immune systems and pregnant women (24). Furthermore, some criminal activities can cause an increased zoonotic risk in the human population. For example, pets that are sold on the black market are not subject to the controls of public health systems and therefore expose buyers to a high zoonotic risk. Often the final destination of pets from illegal trade is families with children, in particular, adolescents.

### Teaching One Health With a Constructivist Psychological Approach: Less Digital, More Action

According to Jean Piaget “*The principal goal of education in the schools should be creating men and women who are capable of doing new things, not simply repeating what other generations have done*” (25). The teachers are not speakers; their role should be of a stimulator of young people's minds not only with theoretical lectures but also by means of letting them do things because “*playing is the child's jo*b” and by acting they discover the world. During the theoretical lesson, we used a few power-point slides since the adolescents suffer from a phenomenon of digital addiction and we favored discussion direct interaction with them, also helped by the presence of utility and pet therapy dogs in the classroom whenever possible. During the practical part, we involved the adolescents into action with the Law Enforcements K9 Units or other utility dog canine Unit depending on the regions or countries involved. The only rule we imposed on ourselves was to deal with all the topics of the questions in the questionnaire during the lessons, because if, as Charles Darwin said before and Stephen Hawkings after, “*Intelligence is the ability to adapt to environmental changes*,” we need to make our adolescents inventors and innovators, capable of doing new things and survive to pandemics, and not simply referring to bad copies of the past generations.

The purpose of this study was to investigate the knowledge and awareness of the One Health concept/Zoonotic risk among adolescents in 6 different countries (Italy, Austria, Slovenia, Germany, Mauritius and Japan).

## Materials and Methods

### The Sample of Students

**Figure 3:**
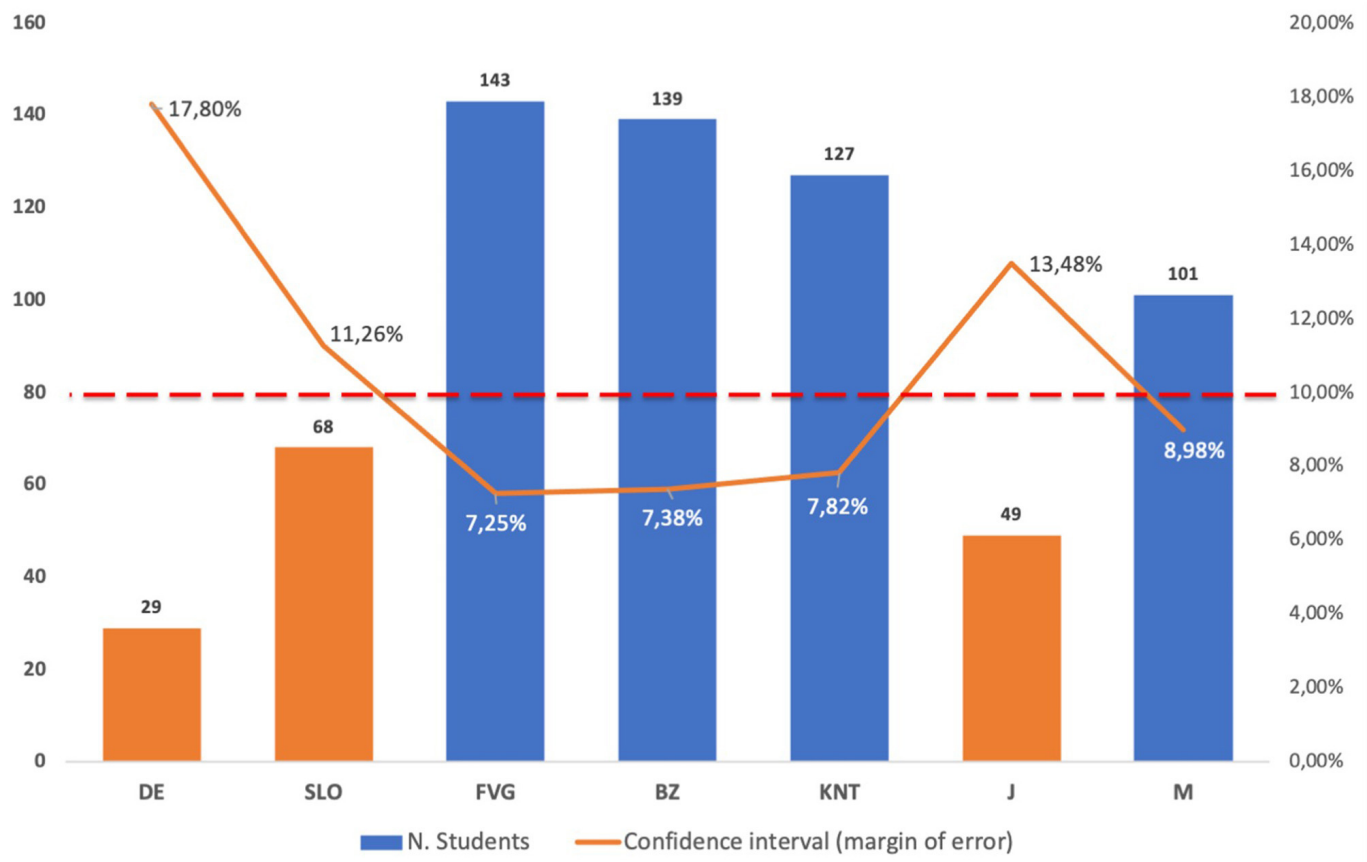
Regions or countries that participated to the research, number of students, and confidence interval (margin of error) of the survey with a confidence level of 95%: DE = Germany (*n* = 29–17.80%), SLO = Slovenia (*n* = 68–11.26%), FVG = Friuli Venezia Giulia Region Italy (*n* = 143–7.25%), BZ = Autonomous Province of Bolzano Italy (*n* = 139–7.38%), KNT = Carinthia, Austria (127–7.82%), J = Japan (*n* = 49–13.48%), M = Mauritius (*n* = 101–8.98%). The confidence level threshold below which the sample was considered not statistically representative is 10%. The sample size with a confidence level of 95% and a confidence interval of 10% with unlimited population size is 97 subjects for each region/country.

The number of adolescents who participated in the survey was 726, but about 10% of the questionnaires were excluded from the analysis because they were incomplete or filled in incorrectly. Therefore, after the exclusion of the questionnaires that did not meet the criteria of correctness, the exact number of the sample under investigation was 656 students. The confidence interval (margin of error) of the survey with a confidence level of 95% has been calculated for each region/country as follows: Germany 17.80%; Slovenia 11.26%, Friuli Venezia Giulia Region 7.25%, Autonomous Province of Bolzano 7.38%, Land Carinthia 7.82%, Japan 13.48%, Mauritius 8.98%. The confidence level threshold below which the sample was considered not statistically representative is 10%. The sample size with a confidence level of 95% and a confidence interval of 10% with unlimited population size is 97 subjects for each region/country. Therefore, the sample of adolescents investigated can be considered statistically representative of the survey for Friuli Venezia Giulia Region, Autonomous Province of Bolzano, Land Carinthia and Mauritius, while Germany, Slovenia and Japan show a confidence interval >10% as reported in Figure 3.

### Countries, Regions, Institutions, and Schools Involved in the Survey

The research project was born at a cross-border level between Italy and Austria in 2018 and then extended to other Italian regions (Autonomous Province of Bolzano), to other European countries (Slovenia and Germany) and extra-European countries (Mauritius and Japan) in 2019. The criterion for the involvement of the European nations involved in the study is related to a broad model of cross-border cooperation between EU countries developed by the Biocrime Center starting from 2017 which not only involves schools but also Law Enforcements and Justice (26). The partners of the Biocrime project took the decision to involve the Republic of Mauritius and Japan as they believed it is essential to acquire comparative information on the perception of biological and zoonotic risk by adolescents living in countries with different climatic, environmental, and cultural situations as compared to Europe. As we are entering the Zoonosecene, a new geological epoch of intensive breeding, of wildlife trade, of antibiotic resistance and of pandemic diseases, following the Anthropocene (7), the only way to overcome a pandemic is to use a global health prevention approach. The institutions that have contributed and participated in the realization of the research project are: Friuli Venezia Giulia Region, Italy; Land Carinthia, Austria; Autonomous Province of Bolzano, Italy; Italian Financial Police, Regional Command of Friuli Venezia Giulia, Italy; Italian Police, Thörl-Maglern International Police Cooperation Center; Carinthian Rescue Dog Unit, Austria; Leibniz Institute for Zoo and Wildlife Research (Leibniz-IZW), Berlin; Mauritius Institute of Education (MIE); Hokkaido University, Japan. The schools that participated in the research project are listed in Table 1.

**Table 1:** Schools that participated in the research project.

Carinthia, Austria	Neue Mittelschulen in Brückl
	Klein St. Paul, Neue MS in St. Veit an der Glan
	BG Villach
	St. Martin Villach
	International School Velden
Friuli Venezia Giulia Region, Italy	I.C. Svevo Trieste
	I.C. Dante Alighieri Trieste
	I.C. Commerciale Corsi Trieste
	I.O. Bachmann Tarvisio
Autonomous Province of Bolzano, Italy	MS Schweitzer Bolzano
	MS Stifter Bolzano
	MS Bozen Stadt Bolzano
Germany	Robert Havemann Gymnasium, Berlin-Karov
Slovenia	Osovna šola Hrpelje
Mauritius	Saint Julien
	Belle Rose
	De La Salle
	New Grove
Japan	Jiyu Gakuen Elementary School

### Age and Gender Ratio

Most of the adolescents involved in the research project were aged between 11 and 13 years (85% of the sample) and the Male/Female sex ratio was balanced with 320 females vs. 336 males.

### The Anonymous Questionnaire

The questionnaire development process had a two-phase approach. Firstly, suggestions from teachers and Law Enforcements were collected to include general aspects of human-animal interaction in the topics of the questionnaire, like the senses, ethology and K9 units. Secondly, the topics that represented the focal point of the survey which relates to health prevention at the human-animal interface were included. The semantics of the terms used in the questionnaire have always been kept very simple with the deliberate exception of the use of the complex term “zoonosis”. The questionnaire consisted of 40 questions, divided into 7 sections: I. Human-animal interface, II. Animal senses, III. Ethology, IV. Microchip and passport, V. One Health, Zoonosis, and diseases, VI. Illegal animals trade, VII. K9 Units and it was translated into Italian, German, English, Slovenian and Japanese languages. This publication deals exclusively with sections I. Human-animal Interface and V. One Health, Zoonoses, and diseases.

**Phase 1 “Naïve” knowledge**: the teachers of the schools administered the questionnaire to the students, without giving them any preliminary lecture on the topics related to the questionnaire in order to assess what was the “prior knowledge” of the adolescents. All the regions and countries involved participated in this phase.

**Phase 2 Learning**: students attended two theoretical and practical lecture sessions at 30-day intervals. Each session consisted of one theoretical lesson of 45 min followed by a practical lesson with utility dog which performed together with the canine Units of Law Enforcements or other utility dog canine Unit depending on the regions or countries involved. During the 30-day intervals adolescents were working with their teachers on these topics. After the second lecture, the same questionnaire of Phase 1 was again administered in order to assess the efficacy of the course and the students' learning rate. Friuli Venezia Giulia Region (Italy), Carinthia (Austria), Slovenia and Germany participated in this second phase.

### Statistical Analysis

A multivariate model with region or country and gender (male, female) as factors has been investigated by means of ANOVA to estimate the prevalence of correct health prevention behaviors and to identify the influential factors for these behaviors.

## Results

### Section I. The Human-Animal Interface

#### Q1: Do You Like Animals? (Yes 96.19%, No 3.84%)

**Figure 4:**
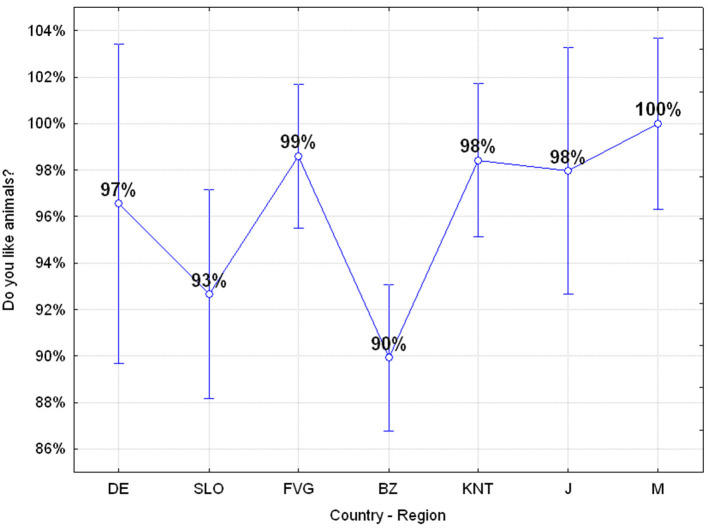
Q1: “Do you like animals?” ANOVA *F*_(6, 649)_ *p* = 0.00023 [Axes X regions or countries, Axes Y % correct answers–DE, Germany; SLO, Slovenia; FVG, Friuli Venezia Giulia Italy; BZ, Autonomous Province of Bolzano Italy; KNT, Carinthia Austria; J, Japan; M, Mauritius–(*n* = 656)].

96.19% of the adolescents like animals although there are significant differences among the different regions or countries. In particular, adolescents from the Friuli Venezia Giulia Region, Carinthia, Japan and in particular from Mauritius are those who like animals more than the adolescents of the other regions or countries involved (Figure 4).

#### Q2: Do You Have an Animal at Home? (Yes 58.99%, No 41.01%)

**Figure 5:**
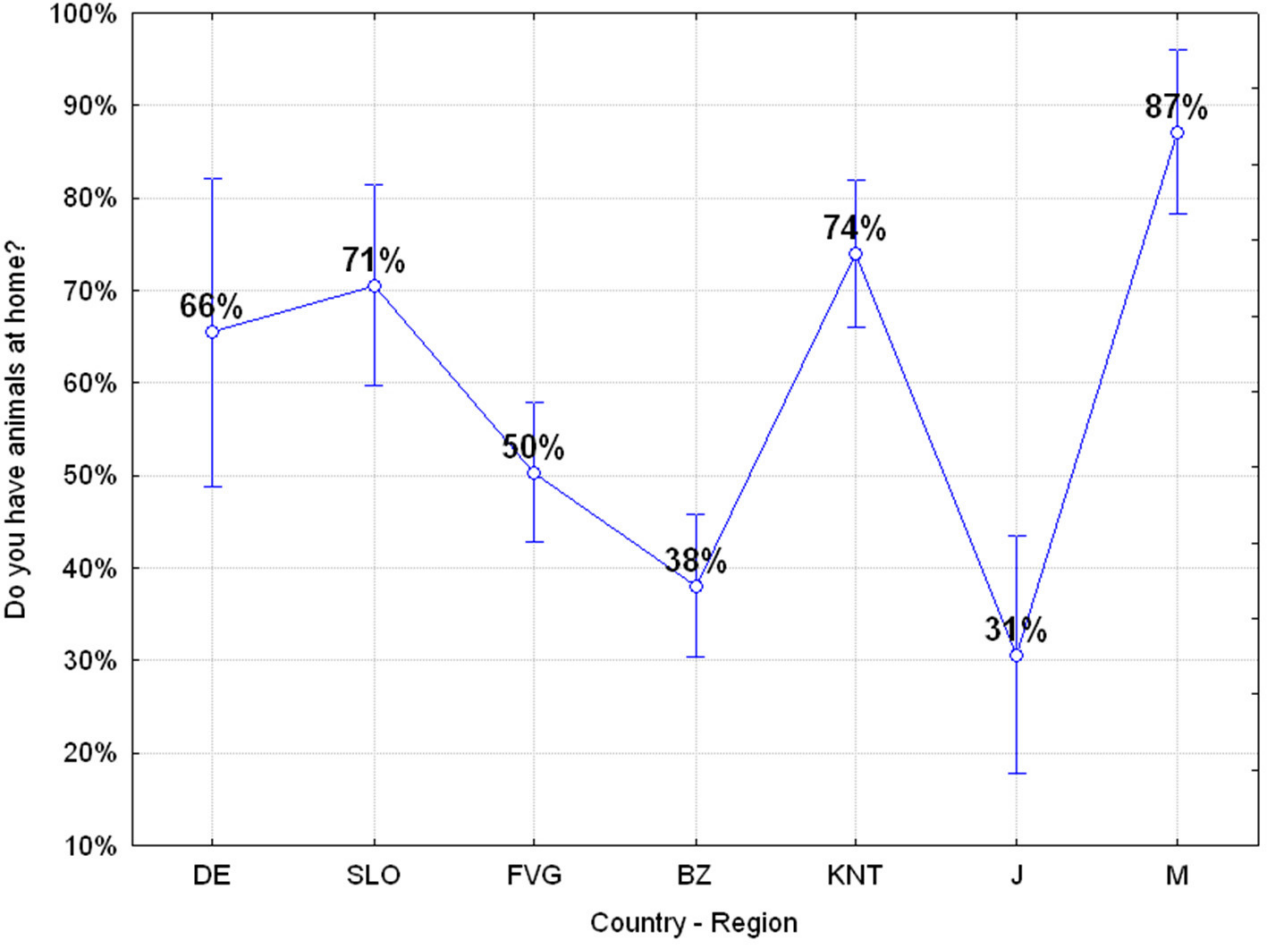
Q2: “Do you have an animal at home?” ANOVA *F*_(6, 644)_ *p* = 0.00000 [Axes X regions or countries, Axes Y % correct answers–DE, Germany; SLO, Slovenia; FVG, Friuli Venezia Giulia Italy; BZ, Autonomous Province of Bolzano Italy; KNT, Carinthia Austria; J, Japan; M, Mauritius–(*n* = 656)].

Exactly 58.99% of adolescents own a pet with the highest percentage values in Mauritian, German, Slovenian, Friuli Venezia Giulia, and Carinthia ones, while in the Autonomous Province of Bolzano only 38% of adolescents claim to have a pet animal. Japanese adolescents have an even lower value but this is probably related to the fact that the survey was carried out in a school located in the urban area of Tokyo (Figure 5).

#### Q3: Are You Afraid of Dogs or Cats? (Yes 7.47%, No 92.53%)

In summary, 92.5% of children are not afraid of dogs and cats and there are no statistically significant differences among the various regions or countries.

#### Q4: Are You Happy That a Dog Is Visiting You at School? (Yes 89.94%, No 10.06%)

Adolescents from all regions or countries are happy that a dog comes to school with a range of average values from 92 to 97% with the exception of Slovenia whose value is significantly lower (79%), ANOVA *F*_(6.630)_ *P* = 0.00136.

### Section V: One-Health, Zoonoses, and Diseases

Results of this section of the questionnaire are summarized and shown in Table 2. Results relating to some of the most significant questions are analyzed in detail below.

**Table 2:** Naïve knowledge–correct answers 1st questionnarie before any lecture–all regions or countries – *n* = 656; Learning–% increasing correct answers 2nd questionnaire after two lectures, DE, Germany; SLO, Slovenia; FVG, Friuli Venezia Giulia; KNT, Carinthia, *n* = 338: Emoticon scale from 5 good (dark green) to 1 very bad (dark red).

**Naïve knowledge** correct answers 1st questionnaire All regions/countries, ***n*** = 656			**Learning** 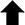 correct answers 2nd questionnaire DE, SLO, FVG, KNT, ***n*** = 338	
**Q.19 “Can pets like dogs and cats become sick like humans?”**				
88.11% correct	11.89% wrong	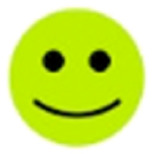	+ 1.92% Learning	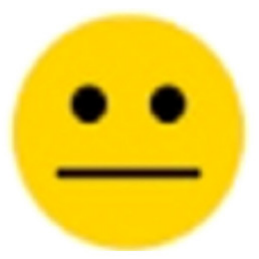
**Q.20 “Must dogs be given a medication against intestinal worms at least once a year?”**				
76.22% correct	23.78% wrong	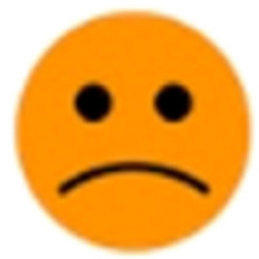	+15.24% Learning	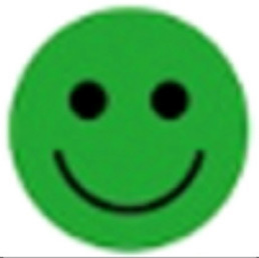
**Q.21 “Is it useful to vaccinate dogs against rabies and other diseases?”**				
91.31% correct	8.69% wrong	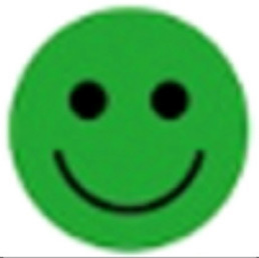	+5.24% Learning	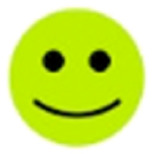
**Q.22: “Many diseases that affect humans come from animals?”**				
71.04% correct	28.96% wrong	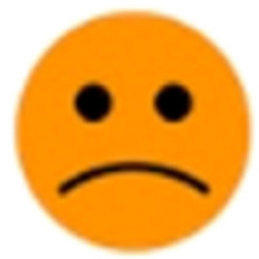	+ 8.49%Learning	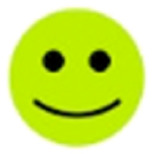
**Q.23: “Zoonoses are diseases transmitted from animals to humans?”**				
67.84% correct	32.16% wrong	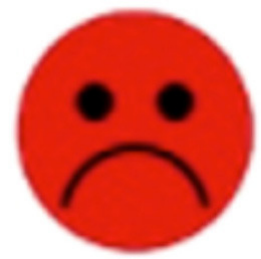	+ 16.98% Learning	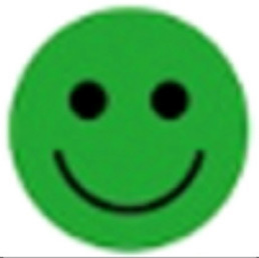
**Q.24: “Can pet animals transmit diseases to humans?”**				
68.60% correct	31.40% wrong	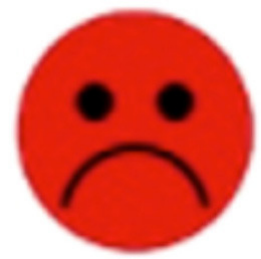	+ 21.92% Learning	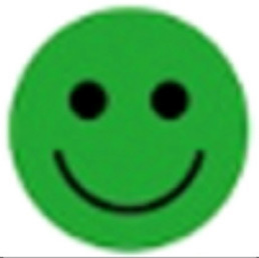
**Q.25: “Can humans transmit diseases to animals?”**				
40.09% correct	59.91% wrong	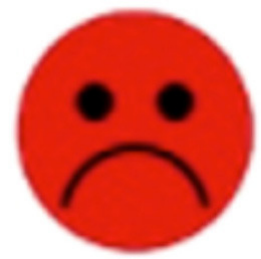	+ 16.39% Learning	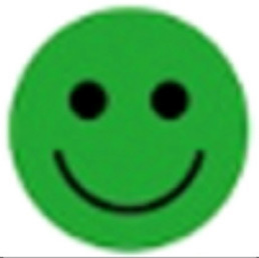
**Q.26: “Is rabies a disease that is dangerous to humans?”**				
76.98% correct	23.02% wrong	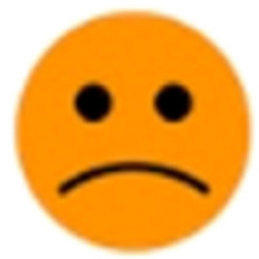	+ 18.07% Learning	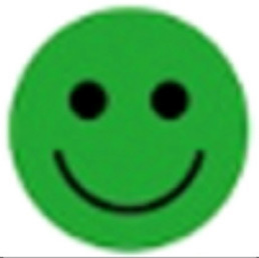

#### Q19: “Can Pets Like Dogs and Cats Become Sick Like Humans?” (88.11% Correct, 11.89% Wrong)

Adolescents have a good understanding of the fact that animals can get sick like people and there are no significant differences between regions or countries and Sex as factors.

#### Q20: “Must Dogs Be Given Medication Against Intestinal Worms at Least Once a Year?” (76.22% Correct, 23.78% Wrong)

**Figure 6:**
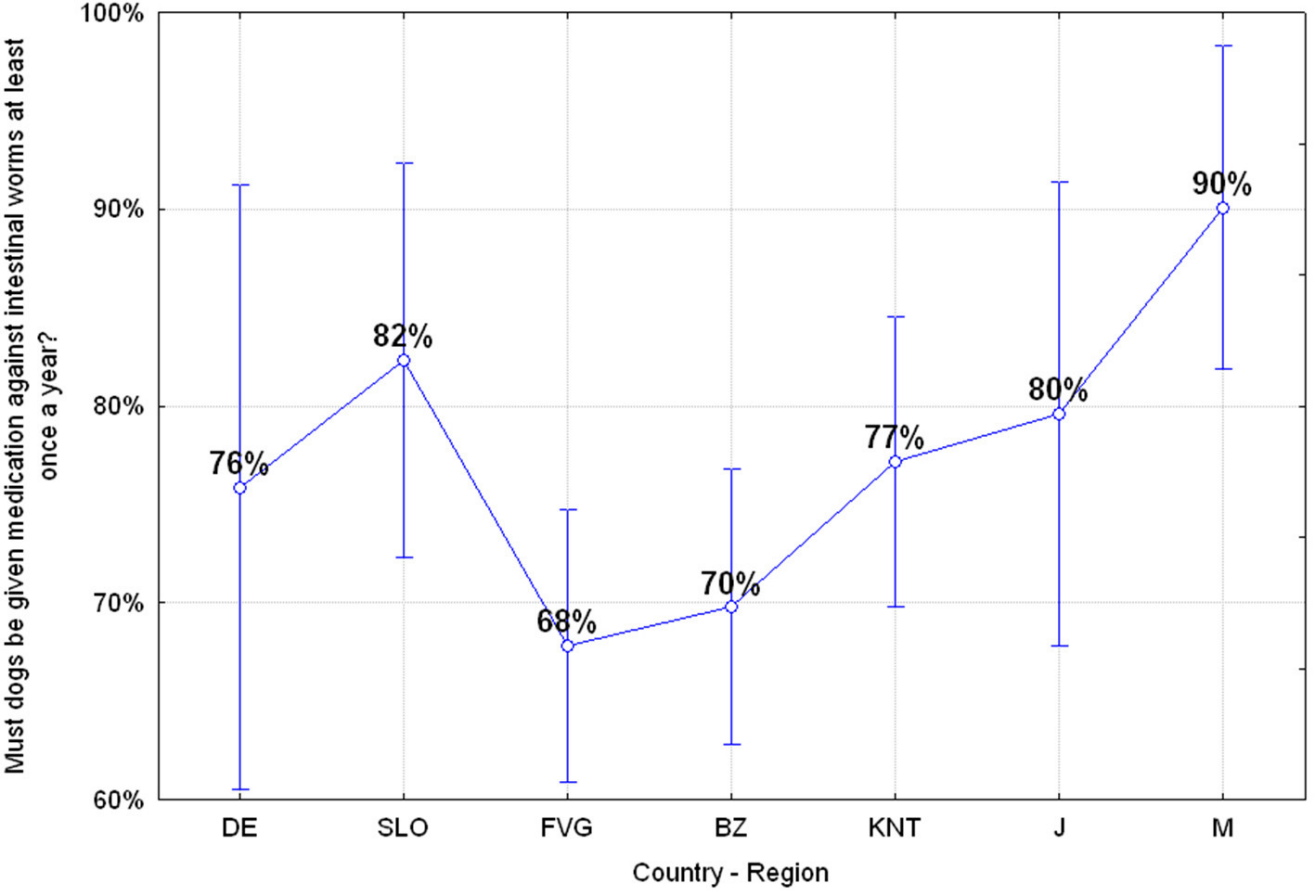
Q20: “Must dogs be given medication against intestinal worms at least once a year?” ANOVA *F*_(6, 649)_ *p* = 0.00153 [Axes X regions or countries, Axes Y % correct answers–DE, Germany; SLO, Slovenia; FVG, Friuli Venezia Giulia Italy; BZ, Autonomous Province of Bolzano Italy; KNT, Carinthia Austria; J, Japan; M, Mauritius–(*n* = 656)].

The percentage of adolescents who gave the wrong answer is almost 24% of the sample. However, there are significant differences among the various regions or countries: adolescents in Mauritius together with the Slovenian ones are those who are most aware about the risk of being infected by intestinal parasites of companion animals ANOVA *F*_(6, 649)_ *p* = 0.00153 as reported in Figure 6. Furthermore, there is also a risk perception bias related to the Sex factor, since females are more aware of this risk, ANOVA *F*_(1, 642)_ *p* = 0.00012.

#### Q21: “Is It Useful to Vaccinate Dogs Against Rabies and Other Diseases?” (91.31% Correct, 8.69% Wrong)

**Figure 7:**
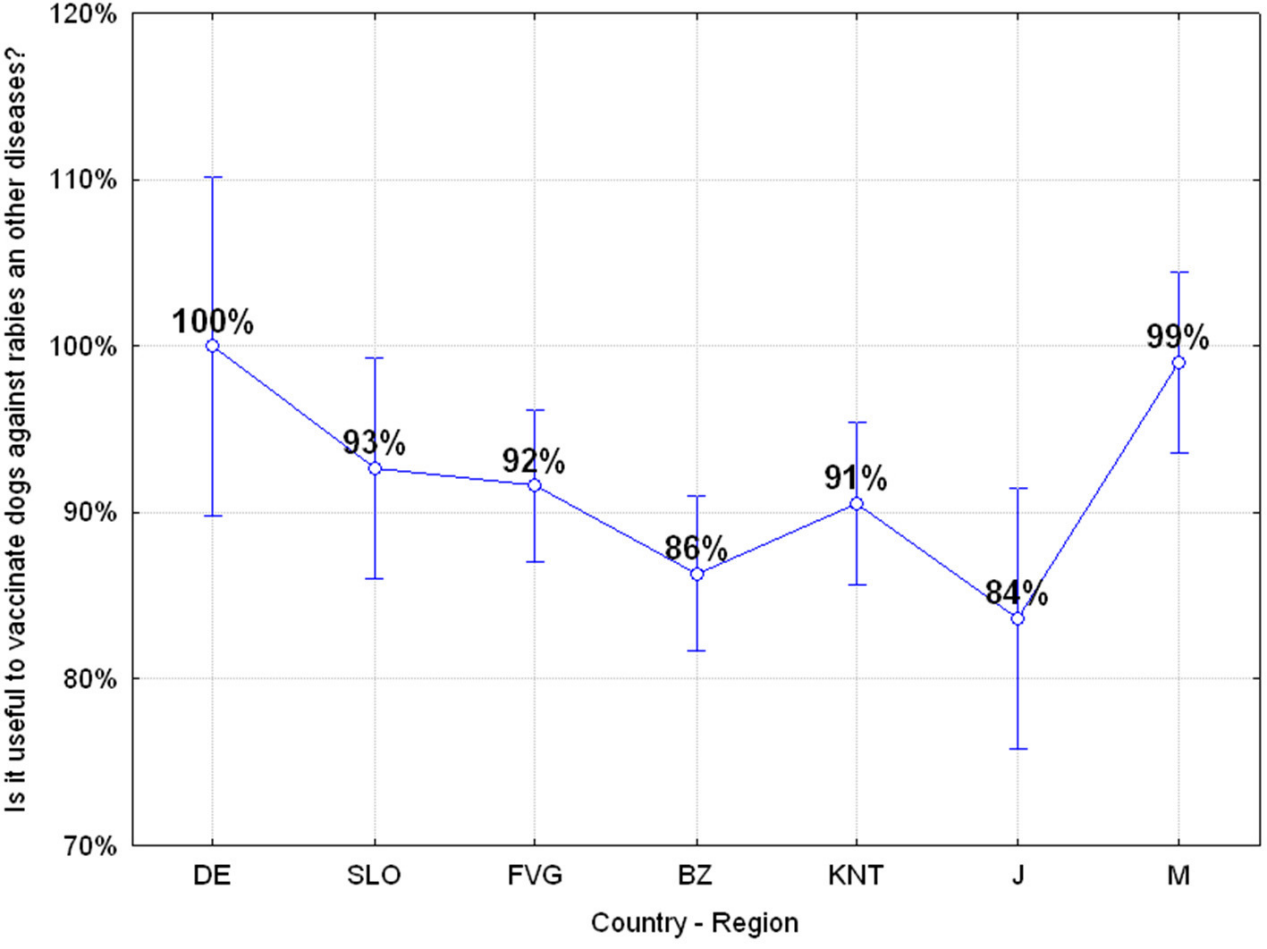
Q21: “Is it useful to vaccinate dogs against rabies and other diseases?” ANOVA F_(6, 649)_ *p* = 0.00482 [Axes X regions or countries, Axes Y % correct answers–DE, Germany; SLO, Slovenia; FVG, Friuli Venezia Giulia Italy; BZ, Autonomous Province of Bolzano Italy; KNT, Carinthia Austria; J, Japan; M, Mauritius–(*n* = 656)].

Adolescents have a good understanding that vaccinating dogs against rabies and other diseases is useful but there are significant differences among the various regions or countries with the German and Mauritian groups showing the greatest awareness and understanding of this risk, ANOVA *F*_(6, 649)_ *p* = 0.00482 as reported in Figure 7. Also, in this case, there is a greater awareness of risk by females compared to males, ANOVA *F*_(1, 642)_ *p* = 0.01247.

#### Q22: “Do Many Diseases That Affect Humans Come From Animals?” (71.04% Correct, 28.96% Wrong)

**Figure 8:**
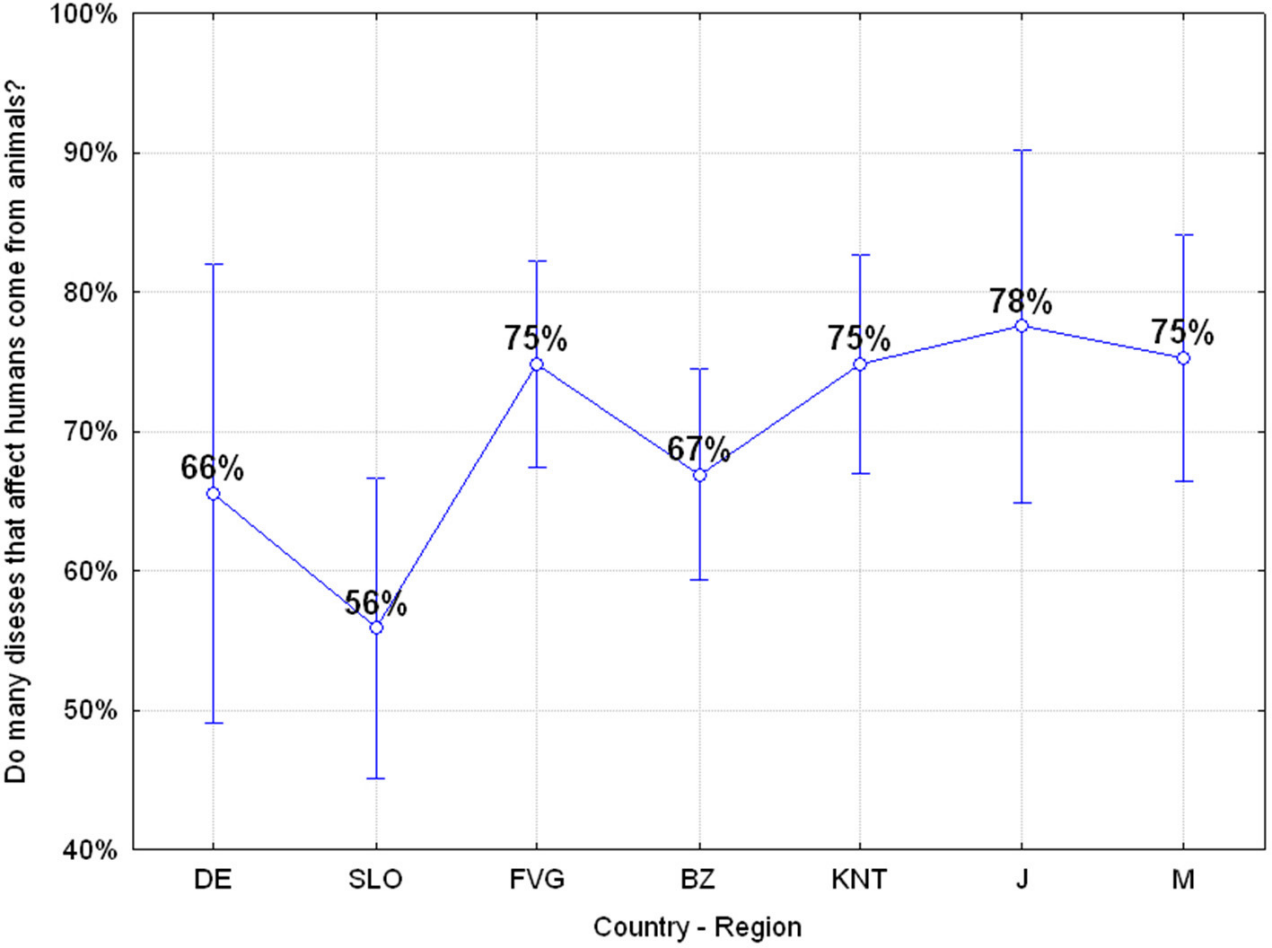
Q22: “Many diseases that affect humans come from animals?” ANOVA *F*_(6, 649)_ *p* = 0.04374 [Axes X regions or countries, Axes Y % correct answers–DE, Germany; SLO, Slovenia; FVG, Friuli Venezia Giulia Italy; BZ, Autonomous Province of Bolzano Italy; KNT, Carinthia Austria; J, Japan; M, Mauritius–(*n* = 656)].

In summary, 28.96% of the adolescents do not perceive the risk of the transmission of diseases from animals to humans and significant differences emerged among the various regions or countries with Japanese and Mauritian adolescents including those of Carinthia and Friuli Venezia Giulia who are more aware of this risk than the others, ANOVA *F*_(6, 649)_ *p* = 0.04374 as reported in Figure 8. Furthermore, Gender as a factor shows that there is a different risk awareness with adolescent males who are more sensitive to this risk ANOVA *F*_(1, 642)_ *p* = 0.02182.

#### Q23: “Do Zoonoses Are Diseases Transmitted From Animals to Humans?” (67.84% Correct, 32.16% Wrong)

**Figure 9:**
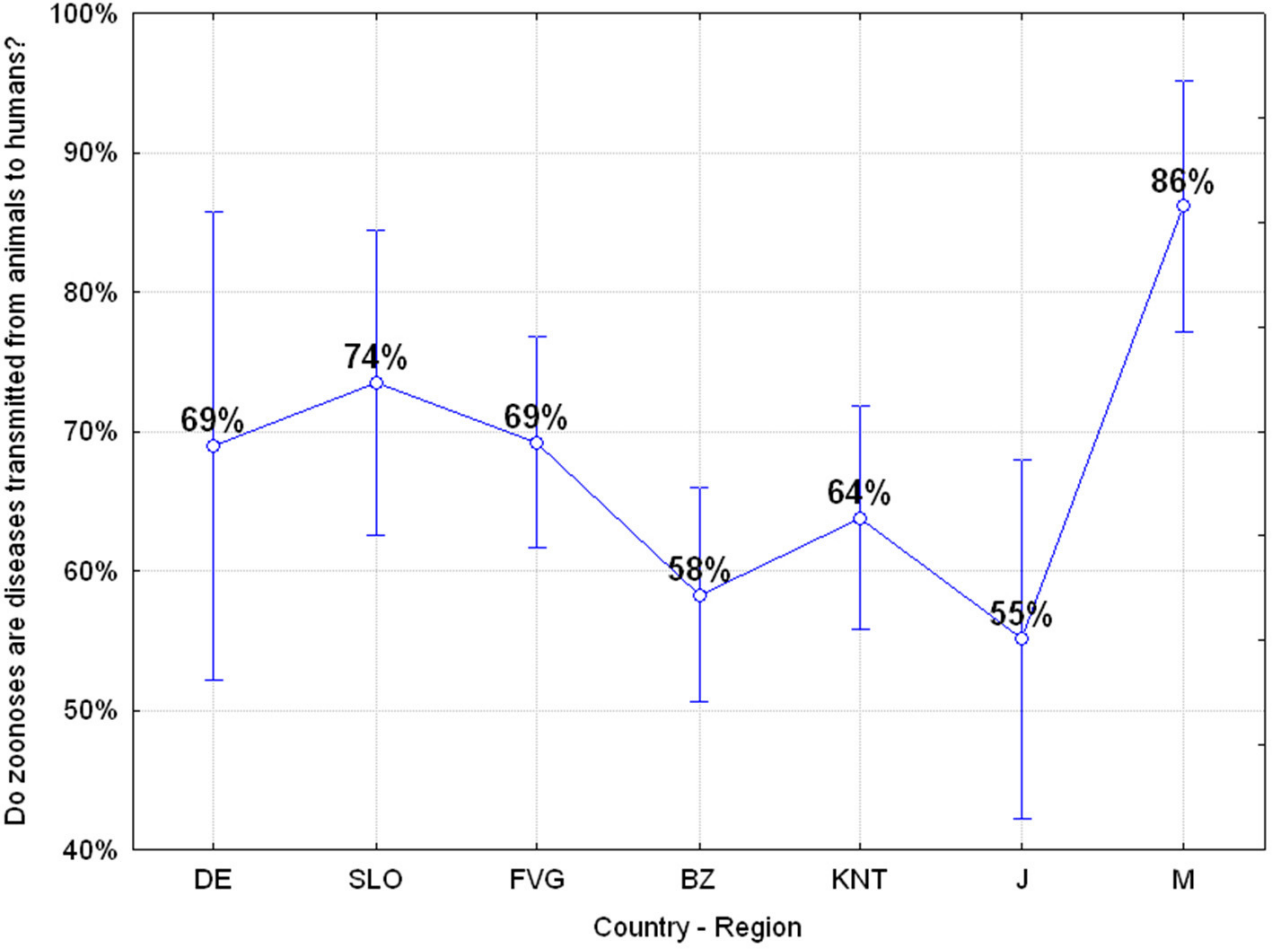
Q23: “Zoonoses are diseases transmitted from animals to humans?” ANOVA *F*_(6, 649)_ *p* = 0.00012 [Axes X regions or countries, Axes Y % correct answers–DE, Germany; SLO, Slovenia; FVG, Friuli Venezia Giulia Italy; BZ, Autonomous Province of Bolzano Italy; KNT, Carinthia Austria; J, Japan; M, Mauritius–(*n* = 656)].

The percentage of adolescents who do not know what zoonoses are is higher than 30% and also in this case substantial differences emerged among the various regions or countries: Mauritian adolescent stand out from all the others for their knowledge of this risk ANOVA *F*_(6, 649)_ *p* = 0.00012, as reported in Figure 9.

#### Q24: “Can Pet Animals Transmit Diseases to Humans?” (68.60% Correct, 31.40% Wrong)

**Figure 10:**
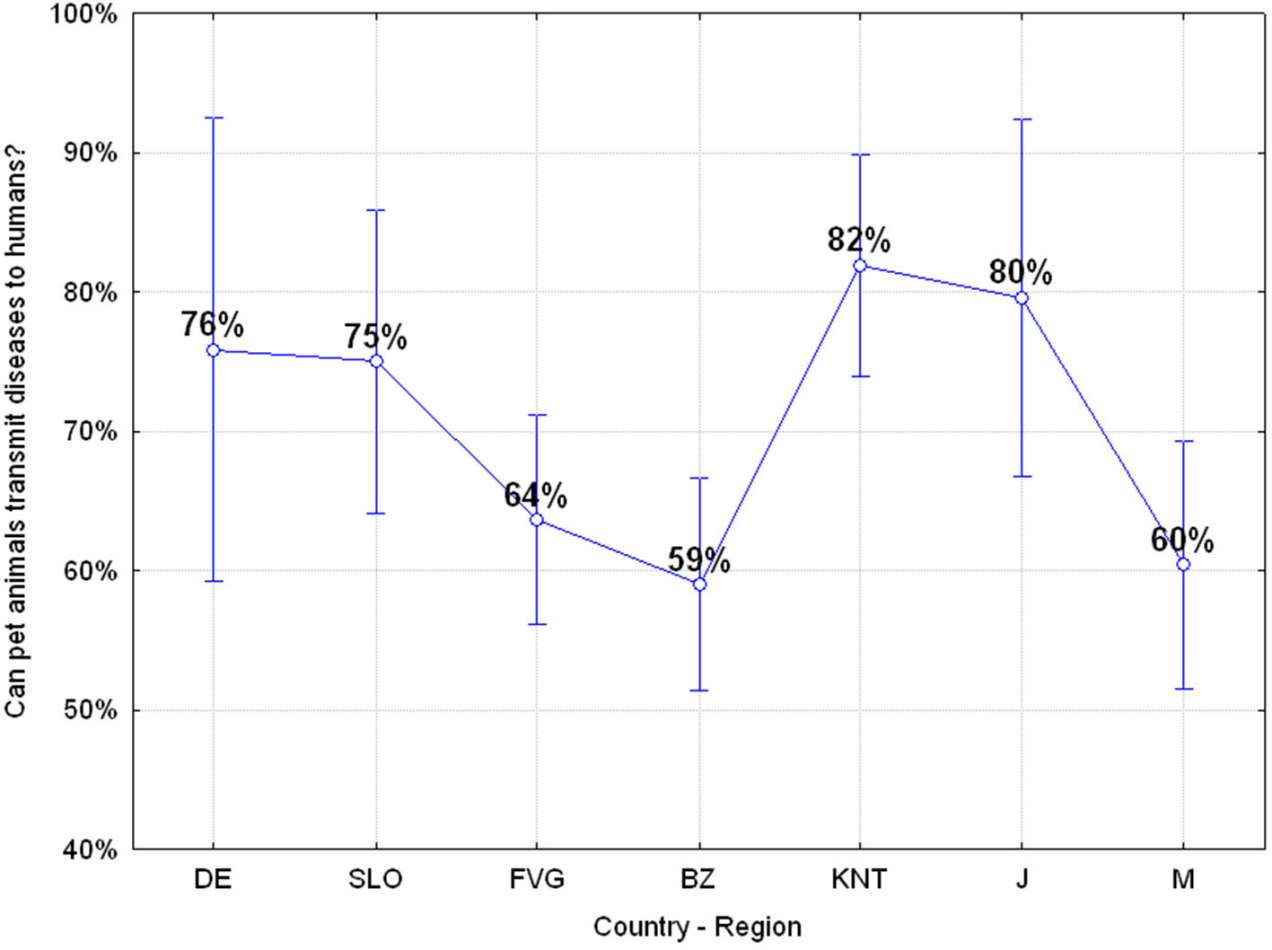
Q24: “Can pet animals transmit diseases to humans?” ANOVA *F*_(6, 649)_ *p* = 0.00020 [Axes X regions or countries, Axes Y % correct answers–DE, Germany; SLO, Slovenia; FVG, Friuli Venezia Giulia Italy; BZ, Autonomous Province of Bolzano Italy; KNT, Carinthia Austria; J, Japan; M, Mauritius–(*n* = 656)].

The results show that 31.40% of the adolescents do not perceive the risk of diseases transmission from pets to humans and significant differences emerged among the various regions or countries with adolescents from Carinthia and Japan being those with the most awareness of this risk ANOVA *F*_(6, 649)_ *p* = 0.00020 as reported in Figure 10.

#### Q25: “Can Humans Transmit Diseases to Animals?” (40.09% Correct, 59.91% Wrong)

**Figure 11:**
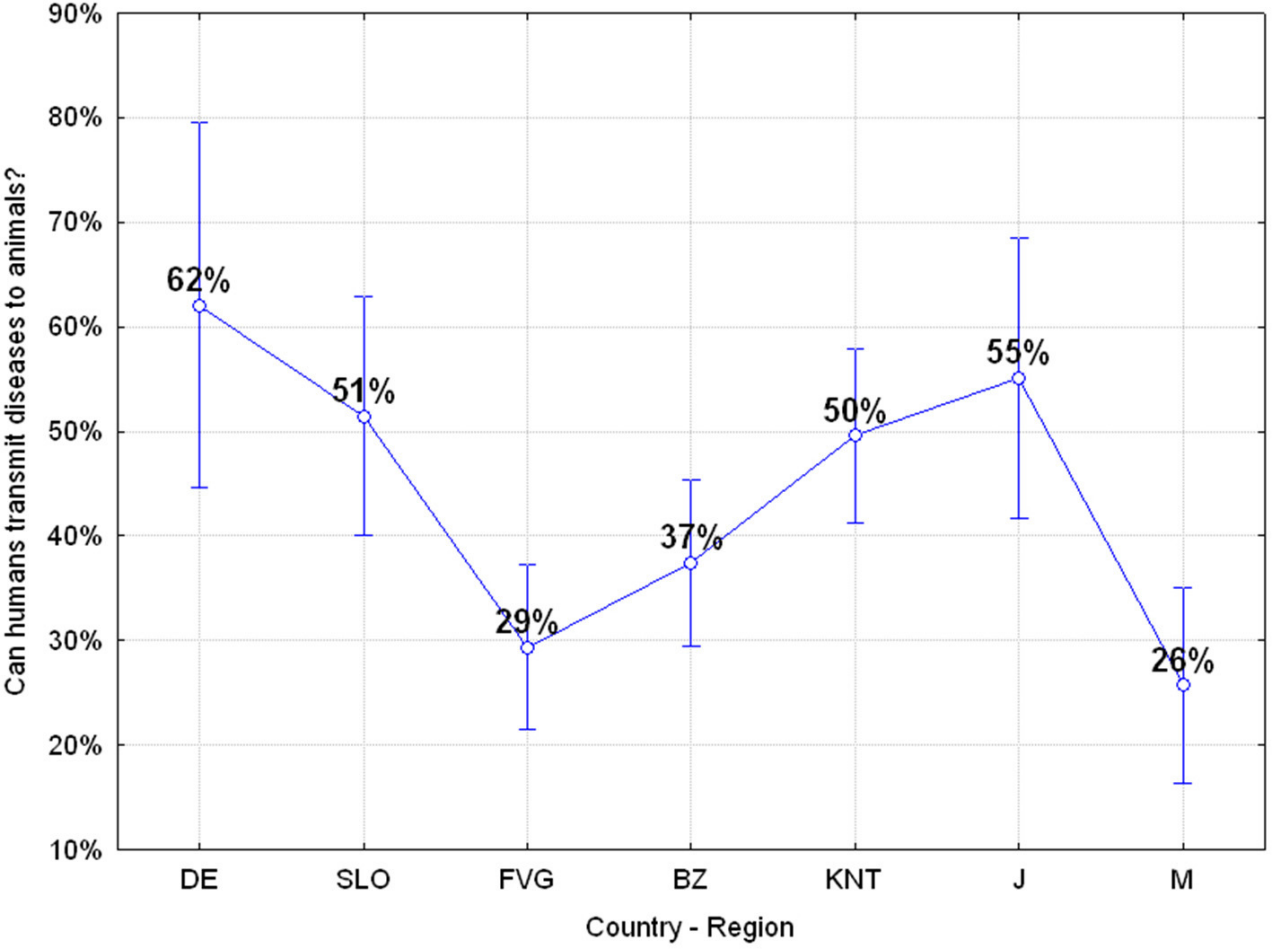
Q25: “Can humans transmit diseases to animals?” ANOVA *F*_(6, 649)_ *p* = 0.00000 [Axes X regions or countries, Axes Y % correct answers–DE, Germany; SLO, Slovenia; FVG, Friuli Venezia Giulia Italy; BZ, Autonomous Province of Bolzano Italy; KNT, Carinthia Austria; J, Japan; M, Mauritius–(*n* = 656)].

Almost 60% of the adolescents believe that humans cannot transmit diseases to animals and also in this case significant differences emerged among the different regions or countries, with the German, Carinthian and Japanese adolescent being more aware of this risk than the others, ANOVA *F*_(6, 649)_ *p* = 0.00000, as reported in Figure 11.

#### Q26: “Is Rabies a Disease That Is Dangerous to Humans?” (76.98% Correct, 23.02 Wrong)

**Figure 12:**
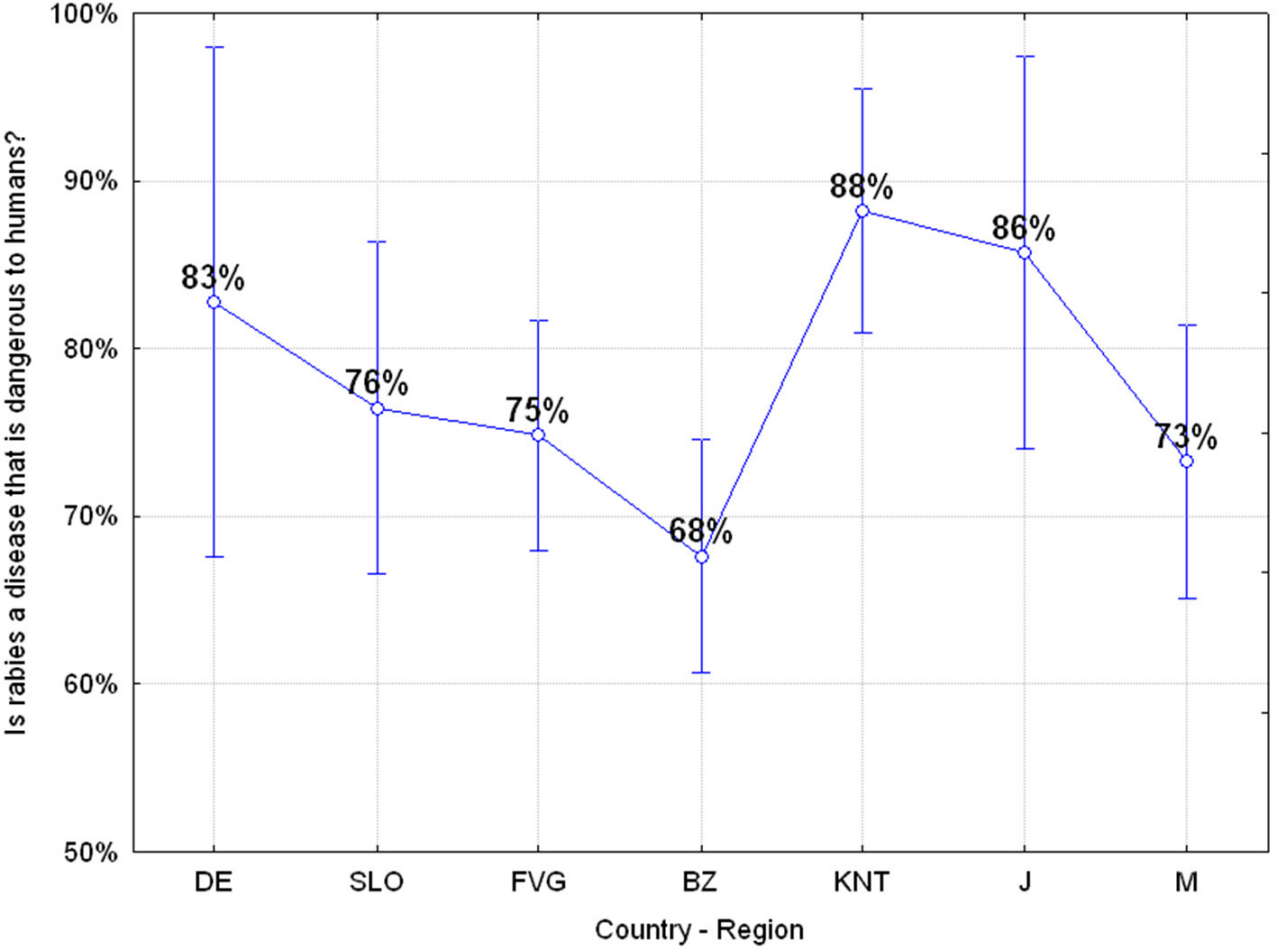
Q26: “Is rabies a disease that is dangerous to humans?” ANOVA *F*_(6, 649)_ *p* = 0.00295 [Axes X regions or countries, Axes Y % correct answers–DE, Germany; SLO, Slovenia; FVG, Friuli Venezia Giulia Italy; BZ, Autonomous Province of Bolzano Italy; KNT, Carinthia Austria; J, Japan; M, Mauritius–(*n* = 656)].

More than 23% of the adolescents believe that rabies is not a dangerous disease for humans and there are significant differences among the various regions or countries with a higher risk awareness among Carinthian, Japanese, and German adolescents compared to others, ANOVA *F*_(6, 649)_ *p* = 0.00295, as reported in Figure 12. Furthermore, we also have an effect of gender as a factor which in particular in Friuli Venezia Giulia and the Autonomous Province of Bolzano determines a significant difference in the risk perception among males and females, ANOVA *F*_(6, 642)_ *p* = 0.00367, with the latest showing the lowest awareness about rabies risks.

## Discussion

### Naïve Knowledge

The Naïve Knowledge of the adolescents shows that they have significant lack of knowledge in the understanding of biological risks and zoonoses. Almost 24% of them are unaware that pets must be dewormed every year (Question 20) and this exposes them to a real risk of being infected by zoonotic parasitic diseases. In fact, most of puppies from the illegal pet trade are tested positive for several endoparasites which are capable of infecting humans such as *Giardia* spp. or *Toxocara* spp. Findings indicate that 67.84% of adolescents know the meaning of the term zoonosis and this is a good result if compared to analogous studies on adults in Brazil where only 28.2% knew the meaning of the term zoonosis (27) or in Portugal where only 35.2% of the adult's group investigated was aware of what is a zoonosis (28).

**Figure 13:**
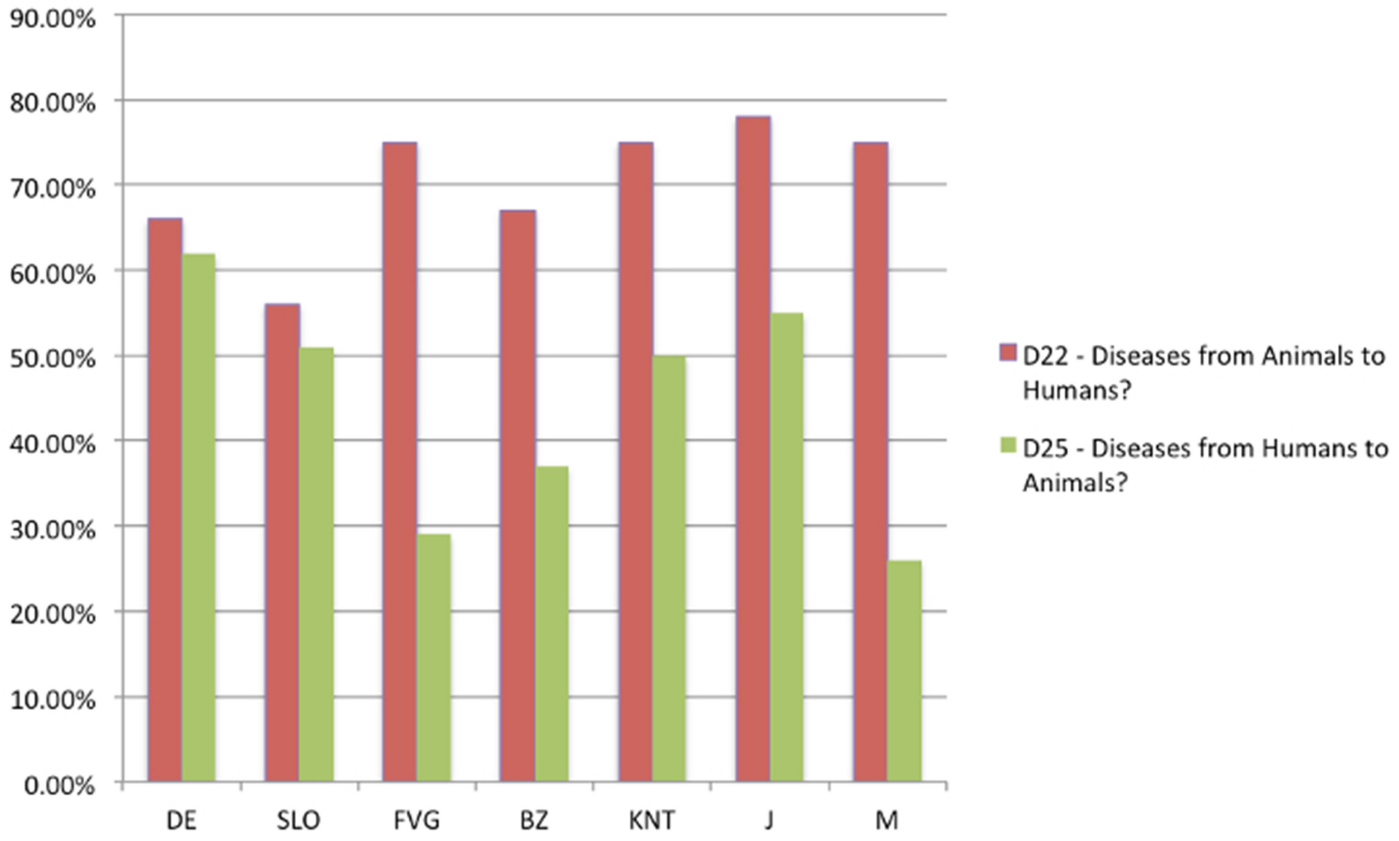
Q22 and Q25: “Many diseases that affect humans come from animals?”–“Can humans transmit diseases to animals?” [Axes X regions or countries, Axes Y % correct answers–DE, Germany; SLO, Slovenia; FVG, Friuli Venezia Giulia Italy; BZ, Autonomous Province of Bolzano Italy; KNT, Carinthia Austria; J, Japan; M, Mauritius–(*n* = 656)].

The survey shows that 31.40% of children do not believe that pets can transmit diseases to humans (Question 24) and this constitutes a dangerous lack of knowledge because it exposes almost one third of the adolescents to a high zoonotic risk. However, the concept that is absolutely the most difficult to understand for the adolescents we investigated is related to the fact whether humans can in turn transmit diseases to animals (Question 25). In this case, 59.91% of the adolescents believe humans are unable to transmit diseases to other species (Figure 13). This lack of knowledge does not allow them to understand properly events such as the reverse-spillover that occurred in Denmark in which human has infected minks with Sar-Cov-2 and minks in turn infected people by generating a new mutation of the Sars-Cov-2 virus named “cluster 5,” which later spread infection to at least eight more countries, namely Denmark, Lithuania, Netherlands, Spain, Sweden, Italy, Greece, and the United States of America (29).

Finally, although 91.31% of adolescents believe it is useful to vaccinate dogs against rabies and other diseases, more than 23% of the same adolescent group believe that rabies is not a dangerous disease for humans. Again, this lack of knowledge exposes them to serious health risks.

### Learning

The usefulness of theoretical and practical lessons in the classroom has its maximum effectiveness in the context of understanding the concept of circularity in the transmission of diseases that can pass from animals to humans and then back again to animals. In this case, only two theoretical and practical sessions in the classroom were enough to generate an increase in knowledge, which varies from a minimum of 16.39% (Question 25) up to a maximum of 21.92% (Question 24) compared to Naïve ones. Nonetheless, the average value of the correct answers among all regions or countries for One Health, Zoonoses and Diseases topics of the questionnaire was 73.88%. This means that 26.12% i.e., more than one quarter of the student population does not understand the basic mechanisms of prevention of infectious diseases with particular reference to zoonoses. It is not only an ethical problem but also a public health problem, which translates into a greater cost to be incurred for the community.

### Limitations of the Study

Though the study was implemented to a comfortable representative sample in four regions or countries (Friuli Venezia Giulia, Autonomous Province of Bolzano, Land Carinthia, and Mauritius) as reported in Figure 3, ideally it would have been better if the pandemic situation permitted, to increase the sample size also in the other three countries (Germany, Slovenia, and Japan) for a broader understanding of the magnitude of the identified associations.

### Future Vision and Hope

The Anthropocene has been a geological epoch characterized by a significant increase of the impact of human activities on the ecosystems. It has been a short evolutionary period because it is obvious to everybody that we have now entered a new period that we could call the Zoonosecene, characterized by the increasingly frequent appearance of pandemic infectious diseases transmitted to human by animals (7). The acquisition of skills and competences in the health prevention and zoonotic domains is of fundamental importance so that the new generations can defend themselves from current and future infectious diseases. The corrective measures to be adopted should start from health prevention programs in schools and from the concept of early education which is the first and most important element of the One Health cycle described in Figure 2. The endless conflict between our species and the zoonoses that can kill us requires the inclusion of the One Health concept in the school's curricula because, as Isaac Asimov said, “*Education is something you can't finish*” like the never-ending war against pathogens.

## Data Availability Statement

The original contributions presented in the study are included in the article/Supplementary Material, further inquiries can be directed to the corresponding author.

## Ethics Statement

Ethical review and approval was not required for the study on human participants in accordance with the local legislation and institutional requirements. Written informed consent to participate in this study was provided by the participants' legal guardian/next of kin.

## Author Contributions

PZu, M-CR, IF, and AB conceived of the idea of developing an international school based survey about One Health and zoonotic risk. PZu, M-CR, MD, YR, TM, SS, SH, AB, IF, GMo, MS, RF, GMe, PZa, FM, and SP contributed to the theoretical lessons. PZu, M-CR, MD, SS, SH, AB, IF, MS, RF, and GMe contributed also the practical lessons with K9 units. PZu wrote the manuscript proof. All authors discussed the results and contributed to the final version of the manuscript.

## Conflict of Interest

The authors declare that the research was conducted in the absence of any commercial or financial relationships that could be construed as a potential conflict of interest.
