# Supplementary material for: What Do Adolescents Know About One-Health and Zoonotic Risks? A School-Based Survey in Italy, Austria, Germany, Slovenia, Mauritius, and Japan
Source: Front Public Health. 2021 Mar 30;9:658876. doi: 10.3389/fpubh.2021.658876 (PMC8045784; doi:10.3389/fpubh.2021.658876)
Supplement: Supplementary file 1 [file Presentation_1.pdf]

frontiers in Public Health

What do adolescents know about One-Health and Zoonotic risks? A school-based survey in Italy, Austria, Germany, Slovenia, Mauritius and Japan

Paolo Zucca, Marie-Christin Rossmann, Mitja Dodic, Yashwantrao Ramma, Toshiya Matsushima, Steven Seet, Susanne Holtze, Alessandro Bremini, Ingrid Fischinger, Giulia Morosetti, Marcello Sitzia, Roberto Furlani, Oronzo Greco, Giulio Meddi, Paolo Zambotto, Fabiola Meo, Serena Pulcini, Manlio Palei, Gianna Zamaro

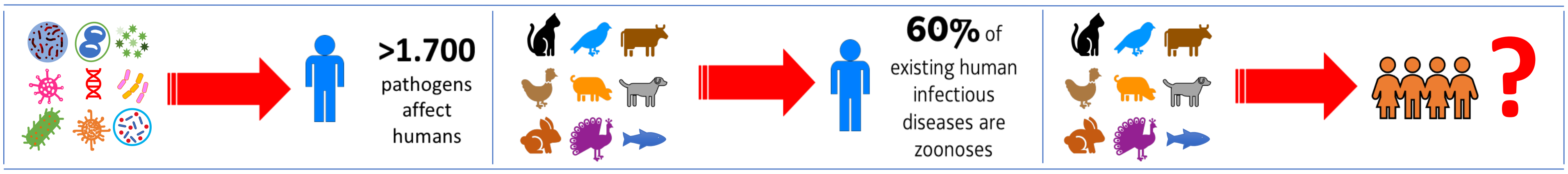

The One Health Cycle explains how the management of a zoonotic spillover with a One Health approach works.

A zoonotic pathogen from wild animals moves into livestock, pets, fur animals or directly to humans. The One Health approach introduces the processes of "**early education**" (green) and "**early prediction**" (blue) that boost the **early detection** (red) and **control** (orange) efforts. Early education increases the number of young people able to recognize a zoonosis, while the **Artificial Intelligence** systems used in early prediction increase the ability to predict an outbreak in wildlife before the spillover can occur. **Anthropogenic pressure, deforestation and bushmeat, climate changes, illegal animal trade and intensive animal farms** are all human-generated causal factors causing the emergence and resurgence of zoonotic diseases.

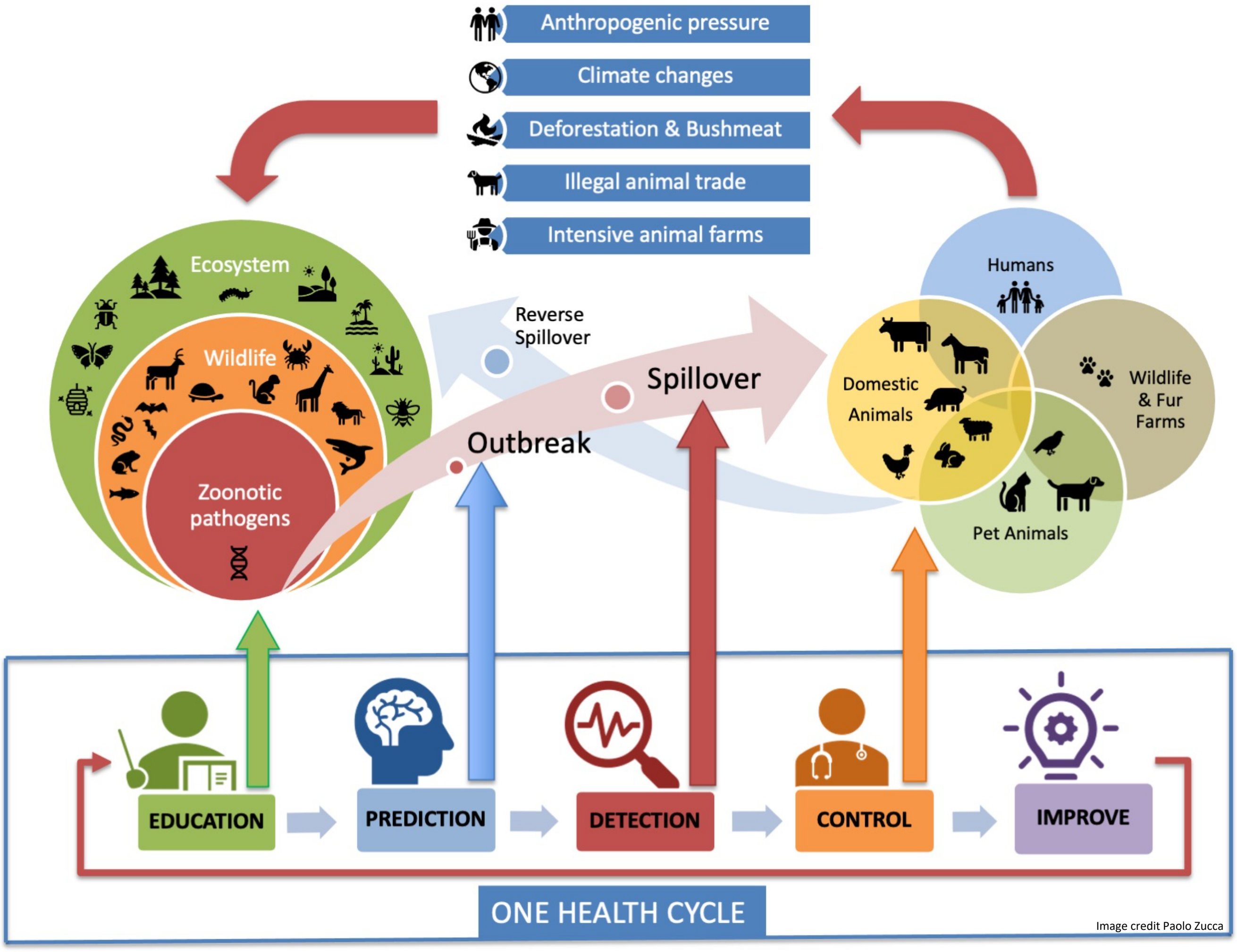

Regions/countries (naïve knowledge): FVG (I), KNT (A), BZ (I), SLO, DE, M, J

NAÏVE KNOWLEDGE<br>correct answers before any lesson<br>1st questionnaire, all regions or countries, n= 656			LEARNING<br>⬆correct answers after two lessons,<br>2nd questionnaire, DE, SLO, FVG, KNT, n=338	
**Q.22: "Many diseases that affect humans come from animals?"**				
71.04% correct	28.96% wrong	☹	+ 8.49% Learning	☺
**Q.23: "Zoonoses are diseases transmitted from animals to humans?"**				
67.84% correct	32.16% wrong	☹	+ 16.98% Learning	☺
**Q.25: "Can humans transmit diseases to animals?"**				
40.09% correct	59.91% wrong	☹	+ 16.39% Learning	☺
**Q.26: "Is rabies a disease that is dangerous to humans?"**				
76.98% correct	23.02% wrong	☹	+ 18.07% Learning	☺

Regions/countries (learning): FVG (I), KNT (A), SLO, DE

Scale: 5 4 3 2 1

The teaching of health prevention with a One Health approach and a practical training should be included in every school curriculum.

Front. Public Health | doi: 10.3389/fpubh.2021.658876
